# *Thymus* spp. Aqueous Extracts and Their Constituent Salvianolic Acid A Induce Nrf2-Dependent Cellular Antioxidant Protection Against Oxidative Stress in Caco-2 Cells

**DOI:** 10.3390/antiox13111287

**Published:** 2024-10-24

**Authors:** Carlos Martins-Gomes, Fernando M. Nunes, Amélia M. Silva

**Affiliations:** 1 Centre for Research and Technology of Agro-Environmental and Biological Sciences (CITAB), Cell Biology and Biochemistry Laboratory, University of Trás-os-Montes and Alto Douro (UTAD), Quinta de Prados, 5000-801 Vila Real, Portugal; amsilva@utad.pt; 2 Chemistry Research Centre-Vila Real (CQ-VR), Food and Wine Chemistry Laboratory, University of Trás-os-Montes and Alto Douro (UTAD), Quinta de Prados, 5000-801 Vila Real, Portugal; fnunes@utad.pt; 3 Department of Chemistry, School of Life Sciences and Environment, University of Trás-os-Montes and Alto Douro (UTAD), 5000-801 Vila Real, Portugal; 4 Department of Biology and Environment, School of Life Sciences and Environment, University of Trás-os-Montes and Alto Douro (UTAD), 5000-801 Vila Real, Portugal; 5 Institute for Innovation, Capacity Building and Sustainability of Agri-Food Production (Inov4gro), University of Trás-os-Montes and Alto Douro (UTAD), Quinta de Prados, 5000-801 Vila Real, Portugal

**Keywords:** cellular antioxidant activity, nuclear factor erythroid 2-related factor 2 (Nrf2), *Thymus carnosus*, *Thymus capitellatus*, salvianolic acid A, Caco-2 cells, oxidative stress, NAD(P)H quinone oxidoreductase (NQO1)

## Abstract

The increasing incidence of colorectal cancer and inflammatory diseases poses a major health concern, with oxidative stress playing a significant role in the onset of these pathologies. Factors such as excessive consumption of sugar-rich and fatty foods, synthetic food additives, pesticides, alcohol, and tobacco contribute to oxidative stress and disrupt intestinal homeostasis. Functional foods arise as a potential tool to regulate redox balance in the intestinal tract. Herbs (such as *Thymus* spp.) have long been screened for their antioxidant properties, but their use as antioxidants for medicinal purposes requires validation in biological models. In this study, we addressed the potential antioxidant protection and preventive effects of extracts from two thyme species at the intestinal level, as well as their molecular mechanisms of action. Caco-2 cells were pre-exposed (4 h) to aqueous (AD) and hydroethanolic (HE) extracts of *Thymus carnosus* and *Thymus capitellatus*, followed by a recovery period in culture medium (16 h), and then treated with *tert*-butyl-hydroperoxide (TBHP; 4 h), before analyzing cell viability. The effect of the extracts’ main components was also analysed. Cellular oxidative stress, cell-death markers, and the expression of antioxidant-related proteins were evaluated using flow cytometry on cells pre-exposed to the AD extracts and salvianolic acid A (SAA). Results showed that pre-exposure to AD extracts or SAA reduced TBHP-induced oxidative stress and cell death, mediated by increased levels of nuclear factor erythroid 2-related factor 2 (Nrf2) protein. The protective activity of *T. capitellatus* AD extract was shown to be dependent on NAD(P)H quinone dehydrogenase 1 (NQO1) protein expression and on increased glutathione (GSH) content. Furthermore, ursolic acid induced cytotoxicity and low cellular antioxidant activity, and thus the presence of this triterpenoid impaired the antioxidant effect of HE extracts. Thus, AD extracts show high potential as prophylactic dietary agents, while HE extracts arise as a source of nutraceuticals with antioxidant potential.

## 1. Introduction

There has been growing awareness of the importance of intestinal barrier homeostasis for overall health, accompanied by trends towards healthier food choices, away from unhealthy lifestyles related to diet or alcohol and tobacco consumption, which are implicated in the increased incidence of intestinal diseases such as colorectal cancer and inflammatory bowel diseases [1,2,3,4,5].

The intestinal epithelium is a semi-permeable barrier, specialized in nutrient and water uptake, forming a boundary between ingested content in the lumen and systemic circulation [6,7]. Nevertheless, this also implies extensive exposure to pathogens, pathogen-derived toxins, and to xenobiotics, such as synthetic food additives, toxicants derived from food processing, pesticides, and others [8]. Examples of pathogen-derived toxins that induce inflammation and oxidative stress in the intestinal tract include lipopolysaccharides [7], deoxynivalenol [9], and ochratoxin A [10]. Among xenobiotics formed during food processing, acrylamide, a carcinogenic molecule, is known to induce oxidative stress and inflammation in the intestinal tract [11,12]. Food additives, such as sweeteners and preservatives, represent another source of oxidative stress in the intestinal tract [13,14], as well as diets rich in sugars and fats [15]. Furthermore, it is known that other food contaminants, such as pesticides, heavy metals, and pharmaceutical drugs induce oxidative stress at the intestinal level [16,17,18,19], highlighting the wide range of oxidative stimuli to which the intestinal tract is exposed.

In the intestinal epithelium, sporadic damage to epithelial cells triggers repair mechanisms that maintain barrier function. However, a persistent oxidative state is known to be responsible for the onset of inflammatory and oncological pathologies [20]. A continuous inflammatory environment leads to the development of chronic inflammatory disorders, such as ulcerative colitis and Crohn’s disease, the most common pathologies associated with inflammatory bowel diseases (IBDs) [21]. In addition to oxidative stress, it is also reported that patients with IBDs present higher risk of developing colorectal cancer [22]. Therefore, countering oxidative stress within the intestinal barrier is a potential strategy to reduce the onset of both inflammation and carcinogenesis. Dietary antioxidant agents are intended to regulate the redox balance in the intestinal tract, either as exogenous antioxidants or as enhancers of endogenous antioxidant systems [23].

Among natural products, herbs and herb-derived products are recognized for their antioxidant properties. Nevertheless, this bioactivity is mostly addressed using chemical colorimetric assays, which primarily measure the ability of samples to scavenge synthetic free radicals. These assays tend to show poor correlation with biological processes [24]. In vivo, the antioxidant activity of phytochemicals is not exclusively dependent on direct radical scavenging, as in most cases the bioactive molecules act as modulators of signalling pathways related to oxidative stress response [25]. Among medicinal herbs, various plants belonging to the *Thymus* spp. have been screened for their free radical scavenging activity. This genus contains species that are currently consumed in human diets, and their extracts have demonstrated free radical-scavenging activity in vitro, as seen with *T. vulgaris* [26] and *T. citriodorus* [26].

However, the antioxidant potential of *Thymus* spp. in biological models, and the relationship between this activity and their phytochemical composition, remain underexplored. This includes *Thymus carnosus* Boiss. and *Thymus capitellatus* Hoffmanns. & Link, two species endemic to mainland Portugal, whose phytochemical composition and radical scavenging activities have been recently described [27,28]. It was reported that both aqueous decoction (AD) and hydroethanolic (HE) extracts of these plants were rich in phenolic acids, namely rosmarinic acid (RA) and salvianolic acids, and in glycoside derivatives of luteolin and quercetin. Additionally, HE extracts presented high amounts of oleanolic (OA) and ursolic acid (UA), and all extracts were shown to effectively scavenge hydroxyl, superoxide and nitric oxide radicals, as well as prevent lipid peroxidation in chemical colorimetric assays [27,28].

Therefore, the main objective of this work was to evaluate the potential of *T. carnosus* and *T. capitellatus* as dietary antioxidant agents with a prophylactic effect against oxidative stress, potentially reducing the incidence of inflammatory and oncological pathologies. To analyse this effect in biological systems, an initial screening was performed using an in vitro model of intestinal epithelial cells, the Caco-2 cell line, and *tert*-butyl hydroperoxide (TBHP) as an oxidative agent. The protection against oxidative stimuli was evaluated in cells pre-incubated with the *Thymus* spp. extracts, with further analysis of oxidative stress markers and key proteins involved in oxidative stress-related signalling pathways. Furthermore, the relationship between the major phytochemicals present in the extracts and their cellular antioxidant activity was evaluated.

## 2. Materials and Methods

### 2.1. Materials

Quercetin, salvianolic acid A, rosmarinic acid, ursolic acid, Mercury Orange (1-(4-chloromercuriophenylazo)-2-naphthol)), propidium iodide (PI), and *tert*-butyl hydroperoxide (TBHP) were purchased from Sigma-Aldrich/Merck (Algés, Portugal). Eriodictyol and oleanolic acid were obtained from Santa Cruz Biotechnology Inc. (Frilabo, Porto, Portugal). Luteolin-7-glucoside was obtained from Extrasynthese^®^ (Genay, France). Cell culture media (Dulbecco’s modified Eagle medium; DMEM), penicillin, streptomycin, foetal bovine serum (FBS), L-glutamine, sodium pyruvate, trypsin-EDTA and versene were obtained from Gibco (Alfagene, Lisboa, Portugal). Alamar Blue^®^ and Hoescht 33342 were purchased from Invitrogen, Life-Technologies (Alfagene, Lisboa, Portugal). Dichlorodihydrofluorescein diacetate (DCFDA), *N*-(fluorescein-5-thiocarbamoyl)-1,2-dihexadecanoyl-*sn*-glycero-phosphoethanolamine (DHPE-FITC) and Annexin-V-FITC were purchased from Thermo Fisher Scientific (Alfagene, Lisboa, Portugal). Anti-Nrf2 (rabbit monoclonal antibody conjugated to phycoerythrin (PE); product number ab223926), anti-HO-1 (mouse monoclonal antibody conjugated to PE; product number ab83214) and anti-NQO1 (rabbit monoclonal antibody conjugated to Alexa Fluor^®^ 488; product number ab196465) were purchased from Abcam (Cambridge, UK). Anti-Phospho-Akt (Ser473) (D9E) XP^®^ (rabbit monoclonal antibody; product number 4060) and anti-rabbit IgG (H+L) F(ab’)2 Fragment (conjugated to Alexa Fluor^®^ 647; product number 4414) were acquired from Cell Signaling Technology, Inc. (Danvers, MA, USA). Other salts and reagents not mentioned above were obtained from Sigma-Aldrich/Merck (Algés, Portugal).

### 2.2. Plant Material, Extracts’ Preparation and Stock Solutions

Cellular antioxidant activity was assessed using AD and HE extracts obtained from *T. capitellatus* and *T. carnosus* aerial parts harvested at the Arrábida National Park in November 2018. All extracts have been previously characterized regarding their phytochemical composition using HPLC-DAD-ESI-MS^n^ [27,28]. Given the near-threatened status of these species, the harvests were previously authorized and supervised by Portuguese Institute for Nature Conservation and Forests (ICNF) (license nos. 867/2018/RECOLHA; 868/2018/RECOLHA). After harvest, the plant material (leaves and stems) was cleaned to remove dirt and debris, rinsed with distilled water, weighed and frozen (−20 °C) for lyophilization (Dura Dry TM P freeze-drier; −45 °C; 250 mTorr).

*T. carnosus* and *T. capitellatus* extracts were obtained as described by Martins-Gomes, et al., 2022 [28]. Briefly, regarding AD extracts, to 0.5 g of lyophilized plant material were added 150 mL of distilled water, and the mixture was heated to 100 °C, under agitation, and held at this temperature for 20 min. After this period, the mixture was allowed to cool down and then filtered using a Whatman no. 4 filter, followed a second filtration using a 1.2 μm fiberglass filter (obtained from VWR International Ltd., Alfragide, Portugal). For HE extracts, to 0.5 g of lyophilized plant material were added 50 mL of a hydroethanolic solution (80% ethanol:20% distilled water; % *v/v*), and the mixture was agitated for 1 h, using an orbital shaker (150 rpm). After this period, the mixture was centrifugated (7000 rpm, Sigma Centrifuges 3–30 K, St. Louis, MO, USA), the supernatant was collected, and the pellet was used to repeat the procedure two more times, for a total of three sequential extraction steps, each with 50 mL of the hydroethanolic solution and conditions mentioned above. The combined supernatants were then filtered (as described above for AD extracts). Using a rotary evaporator (35 °C), both AD and HE extracts were concentrated, step which was also intended to remove ethanol from HE extracts. Each extraction procedure was performed three times for each sample, and all extracts were lyophilized and stored until further use.

Stock solutions (10 mg/mL) were prepared in PBS, for AD extracts, or in 10% DMSO (dimethylsulfoxide; prepared in PBS) for HE extracts. In order to study the association between *T. carnosus* and *T. capitellatus* extracts’ cellular antioxidant activity and their phytochemical composition, the major components of the extracts (identified in Martins-Gomes, et al., 2023 [27], Martins-Gomes, et al., 2022 [28]) were also tested. When a commercial standard of the exact component was not available, the compound with highest structural similarity (e.g., aglycone) was selected. Thus, the compounds selected were rosmarinic acid, salvianolic acid A (SAA; as a representative of salvianolic acids), luteolin-7-*O*-glucoside (L-7-G; as a representative of luteolin glycoside derivatives), quercetin (Q; the aglycone, as representative of quercetin derivatives), and ursolic acid. Stock solutions (10 mM) of all phytochemicals were prepared in DMSO. The final concentration of DMSO in test solutions of extracts and phytochemicals did not exceed 1%, which was previously shown to have no effect on cell viability [29]. Test solutions were prepared from the stock solutions and diluted in FBS-free culture media.

### 2.3. Cell Culture Maintenance and Cell Viability Assessment

The cellular antioxidant activity of *T. carnosus* and *T. capitellatus* extracts, as well as their main components, was evaluated in a cell model of intestinal epithelial cells, Caco-2 cells (obtained from CLS (Cell Lines Service, Eppelheim, Germany)). Cell culture maintenance, handling, subculturing, and seeding were performed as described by Silva, et al., 2020 [30]. For cell viability assays, Caco-2 cells were seeded in 96-well microplates. For flow cytometry, brightfield and fluorescence microscopy, and evaluation of protein levels, Caco-2 cells were seeded in 12-wells microplates. In both cases a density of 5 × 10^4^ cells/mL was used. Prior to the evaluation of *T. carnosus* and *T. capitellatus* extracts protective effect on Caco-2 cells challenged with an oxidative insult, a dose–response (25 to 200 µg/mL) to assess the extracts’ safety profile was performed, and to confirm the safety at lower concentrations. Cell viability was assessed using Alamar Blue indicator, as described by Silva, et al., 2020 [30]. The initial screening was performed for 24 h exposure, as further assays were performed at shorter exposure times (4 h).

For cellular antioxidant activity, concentrations of extracts/compounds that did not induce a reduction in cell viability were selected. The experimental procedure was designed to study the preventive and protective potential of the extracts. Caco-2 cells were pre-exposed to *T. carnosus* or *T. capitellatus* AD and HE extracts at concentrations ranging between 2.5 and 50 µg/mL. The oxidative damage was induced using 250 µM *tert*-butyl hydroperoxide (TBHP) solution, prepared in FBS-free culture medium [31].

The experimental procedure was carried out as follows: Caco-2 cells were exposed to the extracts for 4 h, period after which the test solutions were replaced with FBS-free culture medium, and a new incubation was performed for 16 h. The cells were then challenged with 250 µM TBHP solution (in FBS-free culture medium) for 4 h, period after which the cell viability was assessed through Alamar Blue assay [30].

A second assay was performed following the steps described above, but in which after the oxidative insult, the TBHP solution was removed, and replaced with FBS-free culture medium. The cells were left to incubate for an additional 24 h, recovery period, before assessing cell viability.

To analyse morphological changes, using brightfield microscopy, identical assays were performed. After incubating the cells with 250 µM TBHP for 4 h, the oxidizing agent solution was replaced with PBS, and cell morphology was analysed using an inverted microscope (Lan Optics, Labolan, Esparza, Spain). Images were acquired using a Kern ODC881 Microscope camera (Kern & Sohn GmbH, Balingen, Germany), and acquisition was performed using MicroscopeVIS Image Software (Version 1.0; Kern & Sohn GmbH, Germany).

To assess the cellular antioxidant activity of the main phytochemicals identified in *T. carnosus* and *T. capitellatus* extracts, Caco-2 cells where exposed to 20 µM of SAA, RA, L-7-G, quercetin or UA. A single exposure time of 4 h was considered, and the experimental design was performed as described above. The protective effect of SAA was also evaluated with and without the additional 24 h recovery period.

### 2.4. Evaluation of Oxidative Stress Markers by Flow Cytometry and Fluorescence Microscopy

For flow cytometry assays, Caco-2 cells were pre-exposed to 50 µg/mL of AD extracts of *T. carnosus* and *T. capitellatus*, as well as to 20 µM of SAA, for 4 h, following the procedure described in Section 2.3, both with and without additional 24 h recovery period. After incubation, test solutions were removed, and cells were washed once with PBS, followed by detachment from the microplates, using trypsin-EDTA (~8 min) [32]. After detachment, cells were transferred to microtubes, and centrifuged (bench micro-centrifuge; 3000 rpm; 3 min), the supernatant was removed, and the cells were washed with 600 µL of PBS. The cells were then divided into three equal sets of microtubes to evaluate intracellular ROS levels, glutathione (GSH) content, and lipid peroxidation as previously described by Silva, et al., 2022 [32]. Prior to each assay, cells re-suspended in PBS were centrifuged, the supernatant was removed, and each probe was added to a different set.

One-colour and two-colour assays were performed using a BD Accuri™ C6 cytometer (Becton Dickinson, Franklin Lakes, NJ, USA). In each assay, 10,000 gated events were collected from each sample, and data was analysed using BD Accuri™ C6 Software, version 1.0.264.21 (Becton Dickinson, San Diego, CA, USA).

Briefly, intracellular ROS levels were assessed using dichlorodihydrofluorescein diacetate (DCFDA; Thermo Fisher Scientific (Alfagene, Lisboa, Portugal)). To Caco-2 cells, obtained as described above, 200 µL of DCFDA (10 µM in FBS-free DMEM) were added, followed by a 45 min incubation (37 °C, in the dark). After a washing step (with PBS) to remove excess probe, cells were resuspended in PBS and data was acquired.

Mercury Orange [(1-(4-chloromercuriophenylazo)-2-naphthol); Sigma-Aldrich/Merck (Algés, Portugal)] was used to assess GSH content. Caco-2 cells were treated with 200 µL of 40 µM Mercury Orange solution (in PBS), followed by a 5 min incubation (room temperature, in the dark) and data acquisition.

Lipid peroxidation assessment was performed using DHPE-FITC [*N*-(fluorescein-5-thiocarbamoyl)-1,2-dihexadecanoyl-*sn*-glycero-phosphoethanolamine; Thermo Fisher Scientific (Alfagene, Lisboa, Portugal)]. Cells were handled as described above and then incubated with 20 µM DHPE-FITC solution (in PBS), followed by a 20 min incubation (room temperature, in the dark) and data acquisition.

Intracellular ROS levels were also evaluated using fluorescence microscopy. For this, Caco-2 cells plated in 12-well microplates were treated as described above, and after exposure to TBHP, Caco-2 cells were washed with PBS (37 °C) to remove all TBHP, and then were incubated with 500 µL of DCFDA (10 µM, in FBS-free DMEM) for 45 min (37 °C; in the dark). After incubation, the probe solution was replaced with PBS containing 5 μg/mL of Hoechst 33342 (Invitrogen, Alfagene, Lisboa, Portugal), which was used to assess DNA integrity. After 15 min incubation (37 °C; in the dark), cells were observed using a fluorescence microscope (Olympus IX51; Olympus, Tokyo, Japan) equipped with a DAPI filter (used to observe Hoechst 33342 staining) and FITC filter (used to observe DCF staining), and images were obtained using a coupled CCD camera at Cell^A image acquisition software (Version 2.6, Olympus Soft Imaging Solutions GmbH; exposure time of 20 ms) [33].

### 2.5. Assessment of Cell Death and Evaluation of Oxidative Stress-Response Proteins Using Flow Cytometry

The protective effect induced by *Thymus* spp. extracts and SAA on TBHP-induced oxidative damage was also evaluated as the extracts capacity to prevent cell death. Annexin-V FITC/PI double staining assay was performed as described by Martins-Gomes, et al., 2019 [33]. Briefly, after treatment and detachment, as described in Section 2.4, cells were treated with 200 µL of Annexin V-FITC (1:200 dilution in Annexin-binding buffer [10 mM HEPES sodium salt, 150 mM NaCl, 5 mM KCl, 5 mM MgCl_2_, and 1.8 mM CaCl_2_; pH = 7.4]), and incubated for 20 min, at room temperature, in the dark. After incubation, 5 µL of 50 µg/mL PI (propidium iodide) solution were added, the cells were incubated for an additional 5 min on ice (in the dark), and the data were acquired.

The modulation of p-Akt (phospho-protein kinase B), Nrf2, HO-1 (heme-oxygenase 1) and NQO1 protein levels was assessed by flow cytometry, in fixed and permeabilized cells. After incubating the cells with the extracts, washing, detachment with trypsin-EDTA and its transferring to microtubes as described above, cells were fixed and permeabilized using the methanol permeabilization protocol described for flow cytometry assays, provided by Cell Signaling Technology, Inc. (Danvers, MA, USA) [34]. Briefly, cells were centrifuged to remove PBS and then resuspended in 150 µL of 4% formaldehyde solution, followed by gentle mixing to separate cells and ensure correct fixation. Then the cells were allowed to fix for 15 min at room temperature in the dark. Then, cells were washed twice with PBS (300 µL each wash), and then were resuspended in 100 µL of PBS. For permeabilization, methanol (100%; HPLC grade) previously cooled (−20 °C) was added, in stepwise increments, to the cells suspended in PBS in order to increase methanol concentration up to 90% (total of 900 µL of methanol, for a total volume of 1 mL). This procedure was performed on ice, with gentle mixing of the cells between each addition of methanol. Cells were maintained on ice for 10 min, and then stored at −20 °C until immunostaining.

For antibody staining, fixed and permeabilized cells were washed once with cold PBS (4 °C) and once with room temperature PBS, to remove methanol. Cells were then resuspended in 100 µL of respective antibodies solution, which were diluted in antibody dilution buffer (PBS with 0.5% BSA). Nrf2, HO-1 and NQO1 detection was performed using fluorophore labelled primary antibodies obtained from Abcam (Cambridge, UK), namely: anti-Nrf2 rabbit monoclonal antibody conjugated to phycoerythrin (PE) (1:5000 dilution; product number ab223926), anti-HO-1 mouse monoclonal antibody conjugated to PE (1:2000 dilution; product number ab83214) and anti-NQO1 rabbit monoclonal antibody conjugated to Alexa Fluor^®^ 488 (1:500 dilution; product number ab196465).

Detection of p-Akt was achieved using anti-p-Akt (Ser473) (D9E) XP^®^ rabbit monoclonal antibody (dilution 1:400; product number 4060; Cell Signaling Technology, Inc.) as primary antibody, and anti-rabbit IgG (H+L), F(ab’)2 Fragment conjugated to Alexa Fluor^®^ 647 (1:2000 dilution; product number 4414; Cell Signaling Technology, Inc.) as secondary antibody. Optimization, confirmation of detection and exclusion of autofluorescence influence were analysed for the detection of all antigens.

### 2.6. Data and Statistical Analysis

The results are presented as mean ± SD, based on at least three independent assays. Data and statistical analysis, as well as graphical design, were performed using Microsoft Office Excel (Microsoft Office 365 MSO (version 2409 Build 16. 0. 18025. 20160; Microsoft Corporation, Washington, DC, USA) and GraphPad Prism (Version 8; GraphPad Software Inc., San Diego, CA, USA). For statistical analysis, one-way (single comparison) or two-way (multiple comparison) analyses of variance (ANOVA) followed by Tukey’s multiple test (significance level of 0.05) were performed (GraphPad Prism). Statistical differences (*p* < 0.05) are denoted by different lowercase letters above respective bars in graphs where different conditions are being compared, or by an asterisk (“*”) or hashtag (“#”) when comparing two samples (detailed explanation in each figure legend).

## 3. Results and Discussion

### 3.1. Assessment of Non-Cytotoxic Concentrations and Protection Against TBHP-Induced Cytotoxicity

Antioxidant activity is likely to be the most reported bioactivity for natural products and phytochemicals. However, chemical assays often do not correlate with the antioxidant activity of natural products in animal experimental models. Furthermore, variability between studies can be attributed to the lack of standardization of assays used globally. This standardization would allow an accurate comparison between different publications [24]. To more accurately evaluate the potential of both natural product extracts and their individual phytochemicals in cellular antioxidant activity, cell-based assays have been developed, in which the antioxidant activity was assessed in cells challenged by a standard oxidative agent [35]. Cell-based assays allow for the quantification of oxidative stress biomarkers (e.g., ROS levels, DNA damage, lipid peroxidation), as well as the activity of endogenous antioxidant enzymes, protein expression of oxidative stress-related signalling pathways and various other molecular targets [24,36].

A cell model widely used in studies of antioxidant activity is the HepG2 (human hepatocyte cell model). In HepG2 cells, the cellular antioxidant activity of various dietary natural products (e.g., vegetables or fruits) and their individual phytochemicals has been assessed using oxidative agents such as AAPH [2,2′-azobis (2-amidinopropane) dihydrochloride] and the fluorogenic probe DCFDA as an indicator of cellular oxidative stress levels [35,37,38]. Other oxidative agents, such as hydrogen peroxide (H_2_O_2_) [39] and TBHP [40], have also been employed. Nevertheless, it was proposed that the evaluation of new dietary antioxidants should be addressed in cell models representative of the intestinal tract, such as Caco-2 cells, human intestinal epithelial cells that present normal morphology, functionality and expression of key proteins characteristic of enterocyte [41].

**Figure 1:**
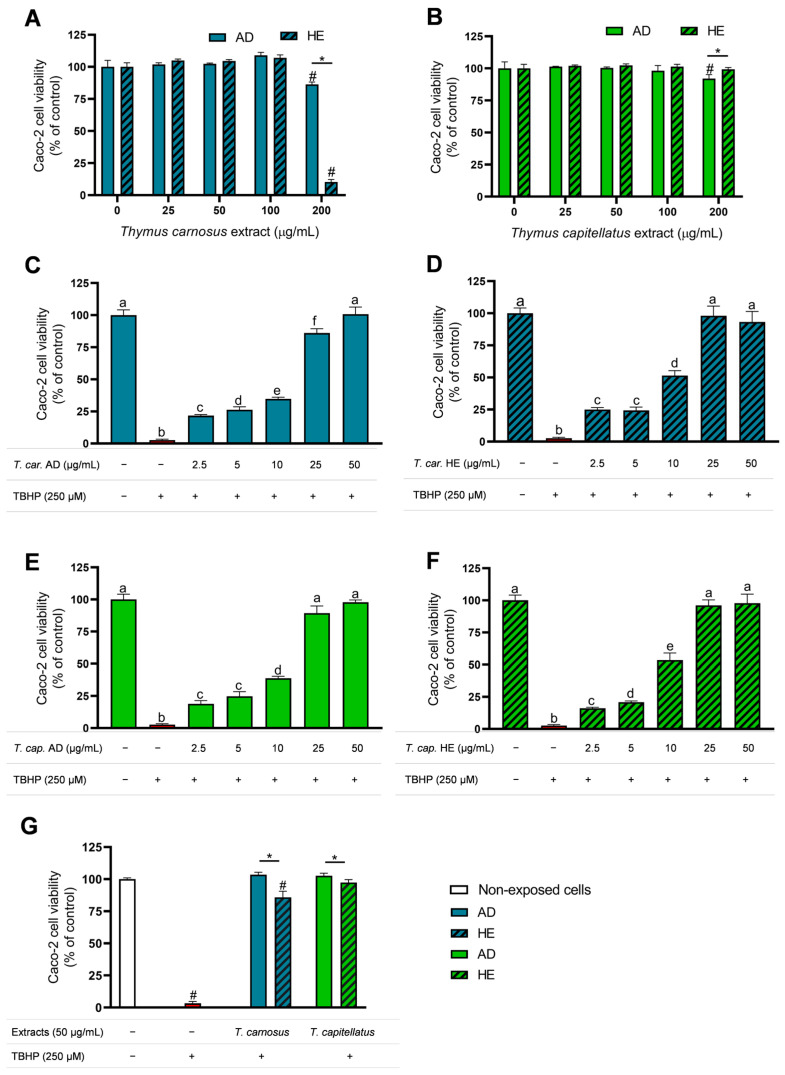
Caco-2 cell viability assays assessing the safety profile of *Thymus* spp. extracts and their protective effect against oxidative stress. A and B: Assessment of the safety profile of *T. carnosus* (**A**) and *T. capitellatus* (**B**) aqueous (AD) and hydroethanolic (HE) extracts using Caco-2 cells (24 h exposure). (**C**–**F**): Assessment of dose-dependent protective effect of extracts against TBHP-induced oxidative damage; cells were pre-exposed to *T. carnosus* (*T. car.*) or *T. capitellatus* (*T. cap.*) extracts (50 µg/mL of AD or HE extracts, as denoted in each panel) for 4 h, then were left to recover culture medium for 16 h, after which were exposed to 250 µM of TBHP (4 h), and then cell viability was evaluated. G: effect of 24 h recovery in culture medium after TBHP challenge assessed in Caco-2 cells subjected to the same procedure as described for panels (**C**–**F**) (see methods for details, Section 2.3). Cells only exposed to 250 µM of TBHP (4 h) are depicted using red bars. Concerning statistical analysis, in panels (**A**,**B**,**G**) significant statistical differences (*p* < 0.05) between samples and the control (non-exposed cells) are denoted as “#” and between extraction methods are denoted with “*”. In panels (**C**–**F**), significant differences (*p* < 0.05) between samples are denoted with different lowercase letters above each bar. Results are presented as mean ± SD (*n* = 3 independent assays).

In the present study, the cellular antioxidant activity of *T. carnosus* and *T. capitellatus* AD and HE extracts was evaluated using Caco-2 cells as an in vitro experimental model, with TBHP as the oxidative agent. To evaluate this bioactivity, the safety profile of the extracts was initially assessed, aiming to select a range of non-cytotoxic concentrations. Results for extracts-induced cytotoxicity and safety profile are presented in Figure 1A and Figure 1B, for *T. carnosus* and *T. capitellatus* extracts, respectively.

As shown in Figure 1A,B, AD and HE extracts of both thyme species only induced statistically significant cytotoxicity at concentrations >100 µg/mL. These results are in agreement with previous data for AD and HE extracts obtained from *T. capitellatus* [28] and *T. carnosus* [29] aerial parts. Nevertheless, Martins-Gomes, et al., 2024 [29] reported that in Caco-2 cells exposed to 100 µg/mL of *T. carnosus* AD and HE extracts, despite not reducing cell viability in Alamar Blue assay, *T. carnosus* extracts induced cell death in Caco-2 cells (Annexin/PI assay). Thus, the cellular antioxidant activity induced by extracts was evaluated for extract concentrations between 2.5 and 50 µg/mL. It was further confirmed, by Annexin/PI assay, that at concentrations up to 50 µg/mL no induction of cell death was observed. Aiming to evaluate the extracts’ potential as prophylactic antioxidant agents, Caco-2 cells were pre-exposed to AD and HE extracts from both thyme species before exposure to the oxidative agent (TBHP), after which cell viability was assessed. The results are presented in Figure 1 (panels C–F).

Pre-exposure of Caco-2 cells to *T. carnosus* and *T. capitellatus* AD and HE extracts significantly reduced TBHP-induced cytotoxicity (*p* < 0.05) in a dose-dependent pattern. As observed, Caco-2 cells pre-exposed to 50 µg/mL AD extracts (of any species), for 4 h, showed high protection against TBHP, with cell viability ≥95% (Figure 1C,E), demonstrating the protective effects of the extracts. At 25 µg/mL, *T. carnosus* HE extracts (Figure 1D) induced higher cellular antioxidant protection compared to AD extracts at the same concentration (Figure 1C). However, in cells pre-exposed to 25 µg/mL or 50 µg/mL of *T. carnosus* HE extracts, the observed cell viability was ~98% and ~93% (*p* > 0.05), respectively, thus a dose-dependent effect is observed only for concentrations up to 25 µg/mL. Comparing HE extracts of both species at 50 µg/mL, a similar level of protection was observed. In *T. capitellatus extracts*, it was found that HE extract between 10 and 25 µg/mL (Figure 1F) provided greater cellular protection than the AD extract (Figure 1E), but at 50 µg/mL, both extracts induced similar cellular antioxidant activity.

Considering all the conditions tested, pre-exposure to 50 µg/mL of *Thymus* spp. extracts for 4 h produced the highest preventive protection against TBHP-induced cytotoxicity (Figure 1C–F). Using these experimental conditions, a second assay was performed on cells that, after exposure to the oxidizing agent (250 µM TBHP; 4 h), were left to recover for 24 h in culture medium, in order to assess whether Caco-2 cells maintained viability. As observed in Figure 1G, no changes were observed in the viability of Caco-2 cells exposed only to TBHP (red bar) compared to the control (non-exposed cells) or to the previous assay (Figure 1C–F). In cells pre-exposed to *T. carnosus* or to *T. capitellatus* AD extracts, a cell viability of ~100% was observed, thus highlighting the cellular antioxidant protection potential of these extracts, whose extraction method mimics typical human consumption of aromatic and medicinal plants and thus more accurately predicts the potential protective effect in in vivo conditions (Figure 1G).

Regarding pre-exposure of cells to 50 µg/mL HE extracts of *T. carnosus* and *T. capitellatus*, initial cell viability immediately after TBHP incubation was ~93% and ~98%, respectively. However, when cells were allowed to recover for an additional 24 h in culture medium, the viability of Caco-2 cells pre-exposed to *T. carnosus* HE extract on average decreased (*p* > 0.05), while cells pre-exposed to *T. capitellatus* HE extract exhibited a similar cell viability (Figure 1G).

**Figure 2:**
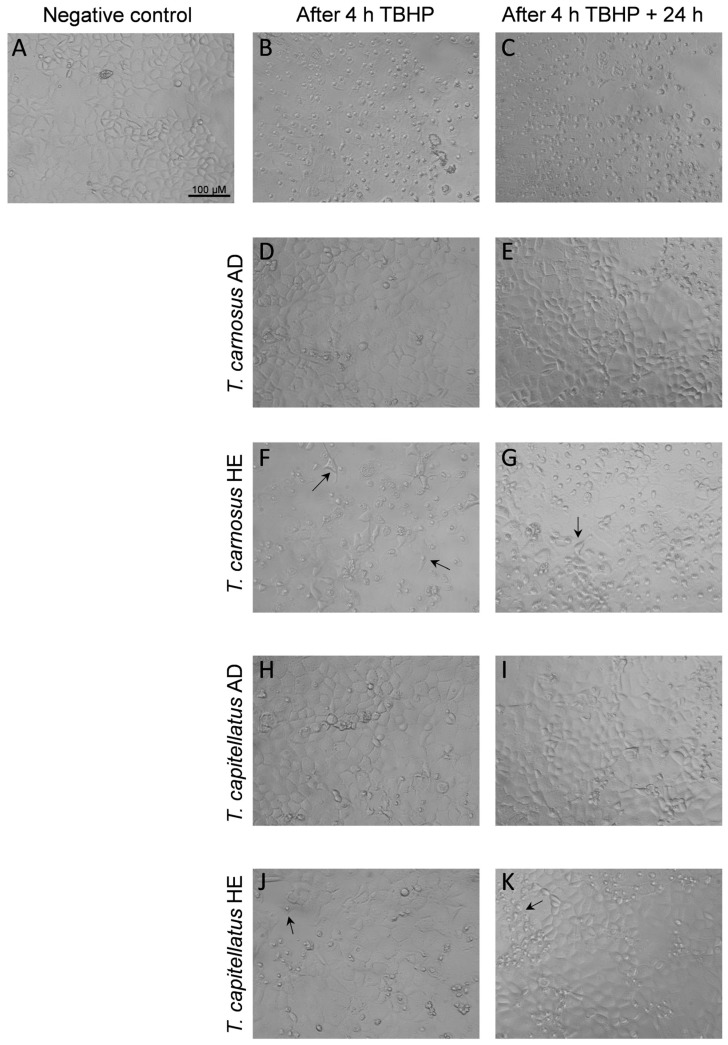
Morphological changes in Caco-2 cells after exposure to TBHP, and the protective effect of *T. carnosus* and *T. capitellatus* extracts. (**A**) negative control, non-exposed cells; (**B**) cells exposed to 250 μM TBHP (4 h); (**C**) cells exposed to 250 μM TBHP (4 h), followed by recovery for 24 h in DMEM. Cells were pre-exposed to 50 μg/mL of extracts for 4 h, followed by 16 h in culture media, and then treated with 250 μM TBHP (4 h) (**D**,**F**,**H**,**J**), or further allowed to recover for additional 24 h in DMEM after TBHP treatment (**E**,**G**,**I**,**K**). Black arrows highlight examples of morphological changes. Images obtained using brightfield microscopy and magnification of 100× (scale bar: 100 μm). See methods for details.

Regarding Caco-2 cell morphology, as observed in Figure 2, control (non-exposed) cells displayed the typical morphology of Caco-2 cells, characterized by a uniform monolayer, with tight cell–cell adhesion and well-defined individual cells. In cells exposed to 250 μM TBHP, significant changes in cell morphology were observed (Figure 2B,C), compatible with the loss of cell viability described above (Figure 1). As seen in Figure 2C, the recovery in culture medium for 24 h after TBHP treatment did not recover the control-like morphology. However, pre-exposure of cells to AD extracts overall prevented TBHP-induced morphological changes, with only a reduced number of cells showing abnormal morphology, and cell density remained similar to the control, thus corroborating the cell viability results.

Cells pre-exposed to HE extracts and then treated with TBHP showed a higher number of cells with morphological changes, characterized by round or elongated shapes and loss of cell–cell adhesion, and lower cell density, when compared to non-exposed cells and to cells exposed to AD extracts. This effect was more pronounced in cells pre-exposed to *T. carnosus* HE extract. In cells that were incubated with culture medium for 24 h after exposure to TBHP, it was observed that cells pre-exposed to AD extracts showed fewer morphological changes, while those pre-exposed to *T. carnosus* HE extract displayed greater damage. Thus, while the two AD extracts and *T. capitellatus* HE extract confer significant cell protection against acute oxidative damage, with the majority of cells maintaining viability and normal morphology 24 h after the oxidative damage (Figure 2E,I,K), in cells pre-exposed to *T. carnosus* HE extracts, a protective effect was observed immediately after TBHP exposure (Figure 1D), but the cells were not able to retain the high viability and morphology after 24 h (Figure 2G).

### 3.2. Evaluation of the Effect of AD Extracts from Thyme spp. and SAA on TBHP-Induced Oxidative Stress and Cell Death

**Figure 3:**
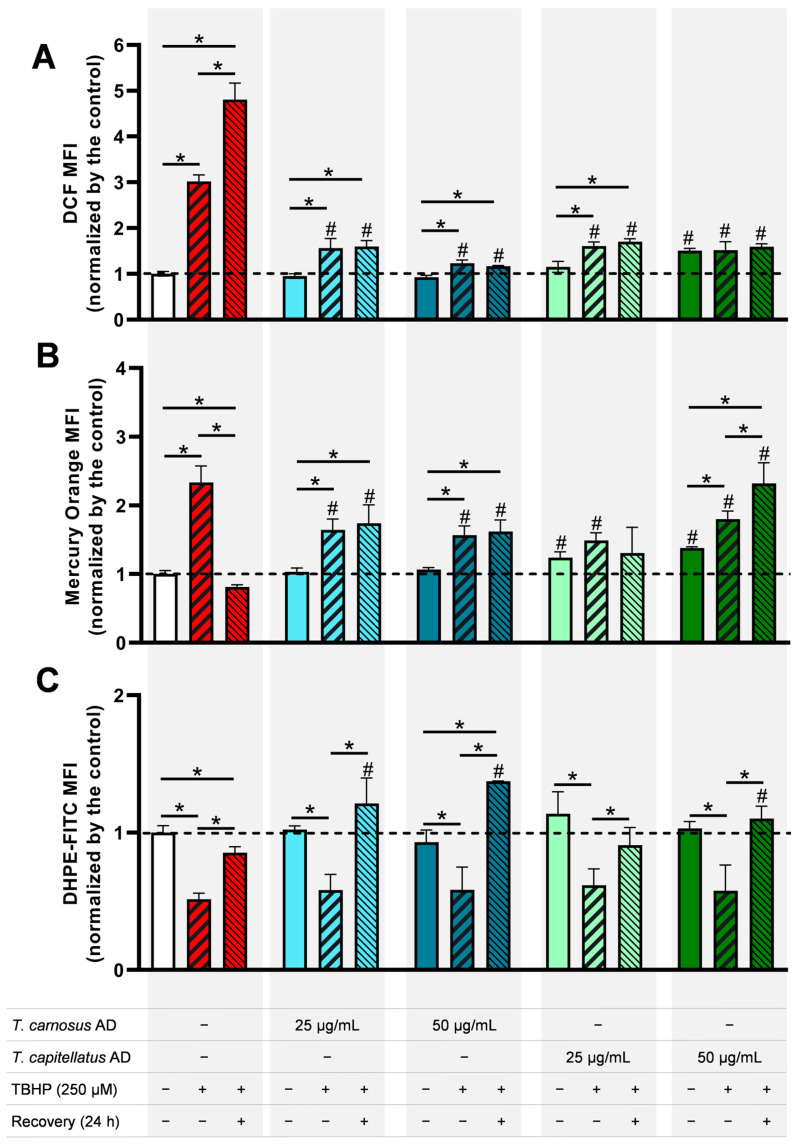
Assessment of oxidative stress markers in Caco-2 cells. Cells were pre-exposed to extracts for 4 h, allowed to recover for 16 h before being exposed to TBHP (250 μM TBHP), or were let to recover in culture medium for additional 24 h before analysis, as denoted. Flow cytometry assessment of intracellular ROS (**A**), GSH content (**B**) and lipid peroxidation (**C**). MFI was normalized to control (non-treated cells), in each assay. MFI: mean fluorescence intensity. Significant statistical differences (when *p* < 0.05) samples and respective control were denoted with “#”. Significant statistical differences (when *p* < 0.05) between different treatments (no TBHP; 4 h TBHP; 4 h TBHP and 24 h recovery) for each sample were denoted “*” over a horizontal bar. All data was normalized to negative control (dotted line). Results are presented as mean ± SD (*n* = 3 independent experiments).

Since the results presented in Figure 1 and Figure 2 showed that AD extracts have higher cellular protective effects against oxidative damage, AD extracts from *T. carnosus* and *T. capitellatus* were selected to further assess their effect on markers of oxidative stress in Caco-2 cells challenged by oxidative insult. Intracellular ROS content and GSH levels, along with lipid peroxidation, were assessed using flow cytometry. Figure 3A presents intracellular ROS levels. As shown, Caco-2 cells exposed to the oxidative agent TBHP (250 μM, 4 h) significantly increased intracellular ROS levels (*p* < 0.05), with this effect enhancing 24 h after exposure to TBHP. In Caco-2 cells pre-exposed to the extracts, it was observed that *T. carnosus* AD extract per se did not induce oxidative stress (i.e., did not change intracellular ROS content, compared to the negative control), but was effective in protecting cells against TBHP insult as it significantly reduced TBHP-induced ROS increase. This effect was dose-dependent, as, on average, cells pre-exposed to *T. carnosus* AD extract at 50 μg/mL present lower ROS levels when compared to those pre-exposed to 25 μg/mL, supporting the dose-dependent increase in cell viability observed in Figure 1C. Intracellular ROS levels remained low 24 h after the oxidative stimulus for both concentrations tested.

At 25 μg/mL, the AD extract of *T. capitellatus* produced a similar effect to that described for *T. carnosus* AD extract. However, pre-exposure of cells to 50 μg/mL *T. capitellatus* AD extract, for 4 h, increased basal intracellular ROS levels, compared to non-exposed cells, but still maintained the ability to protect against TBHP-induced ROS elevation (Figure 3A). In this last condition, *T. capitellatus* AD extract at 50 μg/mL, the concept of hormesis can justify the results obtained.

A protective activity via hormesis is commonly achieved by exposing cells to a non-cytotoxic dose of a toxic agent or factor, stimulating an adaptative behaviour that promotes the build-up of defence and survival mechanisms, allowing cells to later endure higher doses [42]. In this case, the non-cytotoxic increase in intracellular ROS induced by *T. capitellatus* AD extract may stimulate an antioxidant response in Caco-2 cells, allowing them to overcome TBHP-induced oxidative damage. It was found that pre-exposure to the extracts prevented the increase in ROS induced by TBHP (Figure 3A, green bars). This mechanism was described in cells exposed to H_2_O_2_ [42]. At low doses, H_2_O_2_ triggers antioxidant and cell survival pathways, conferring protection against high doses of H_2_O_2_; and also activates Nrf2 pathway that leads to the production of antioxidant enzymes and GSH [42]. In our assay, pre-exposure of cells to extracts for 4 h is followed by a 16 h period in culture medium prior to the oxidative challenge. During this period, the adaptative response of Caco-2 cells induced by extracts may result in increased production of intracellular antioxidant factors.

Among oxidative stress biomarkers, GSH is one of the most relevant intervenient in oxidative stress management [43]. Cellular response to oxidative stress often includes an increase in GSH content, while GSH depletion is commonly associated with cell death [43,44]. Therefore, to understand the cellular antioxidant action induced by extracts, GSH content in Caco-2 cells was measured, and results are presented in Figure 3B. A significant increase in intracellular GSH content was observed in Caco-2 cells exposed to TBHP (250 μM). However, when measured 24 h after exposure to TBHP a reduction in GSH content was observed (Figure 3B), consistent with GSH depletion and consequent cell death. Considering the potential hormetic effect, pre-exposure of cells to 50 μg/mL of *T. capitellatus* AD extracts, which per se induced a slight increase in intracellular ROS (Figure 3A), also induced a significant increase in GSH content (Figure 3B). In cells pre-exposed to *T. carnosus* AD extract, but not exposed to TBHP, GSH content did not change. Although *T. carnosus* AD extract per se did not increase basal levels of GSH, it did protect cells by exerting an antioxidant effect, since the increase in GSH induced by TBHP was significantly attenuated (Figure 3B), reflecting the protection against TBHP-induced ROS (Figure 3A). This protection was long-lasting because high levels of GSH remained even after the 24 h recovery period, indicating that the extract prevented GSH depletion.

Another biomarker of oxidative stress is lipid peroxidation. TBHP was reported to induce lipid peroxidation (evaluated as malondialdehyde content) in Caco-2 cells, although at a higher concentration (2.5 mM; [45]) than that used in the this work (250 μM). Figure 3C shows the results of lipid peroxidation in Caco-2 cells pre-exposed to extracts and then treated with TBHP, using flow cytometry and DHPE-FITC fluorescent probe. Lipid peroxidation is inversely correlated with the MFI of DHPE-FITC, as the initial form of the probe is fluorescent, but products of lipid peroxidation cleave the FITC moiety, resulting in the non-fluorescent form (DHPE) [32]. Thus, samples with MFI < 1 indicate lipid peroxidation higher than that observed under basal conditions (non-exposed cells, negative control). As seen in Figure 3C, 4 h of exposure to 250 μM TBHP induced lipid peroxidation in Caco-2 cells (1.96-fold increase), which was reduced after the recovery period (1.18-fold increase). It was also found that pre-exposure to any of the extracts was not effective in reducing TBHP-induced lipid peroxidation in the cells analysed after exposure to TBHP (*p* > 0.05), although the average levels of lipid peroxidation are lower than the respective control. However, when cells were allowed to recover for 24 h, lipid peroxidation values are similar or lower than that of negative control cells. None of the tested extracts induced lipid peroxidation in Caco-2 cells (Figure 3C). The elevated GSH levels observed in Figure 3B must have contributed to the reduction in lipid peroxidation now observed (Figure 3C).

The cellular antioxidant activity induced by various natural products has been previously reported. In Caco-2 cells, pre-exposure to elderberry extracts (50 μg/mL) was shown to protect against 250 μM TBHP-induced oxidative damage, resulting in cell viability between 60% and 70%, whereas 250 μM TBHP alone reduced cell viability to 19% [31], a lower protection than that exerted by *Thymus* extracts (Figure 1). Elderberry extracts also reduced intracellular ROS levels, maintained control-like GSH content, and reduced lipid peroxidation [31]. Also, Caco-2 cells exposed to an aqueous extract of the fungus *Engleromyces goetzei* (at 1, 5 and 10 mg/mL; 12 h exposure) showed protection against the cytotoxicity induced by TBHP (2 mM; 5 h exposure), and the extract effectively reduced ROS levels and lipid peroxidation [46].

Regarding herbs, Lima, et al., 2007 [47] reported the protective effects of water and methanolic extracts of *Salvia officinalis* against TBHP-induced cytotoxicity in HepG2 cells. At concentrations ranging from 10 to 100 μg/mL, the extracts prevented TBHP-induced cell death, lipid peroxidation and GSH depletion [47]. Also in HepG2, a *Perilla frutescens* aqueous extract (at 1 mg/mL) prevented cytotoxicity, GSH depletion and lipid peroxidation induced by 300 μM TBHP [48]. This effect was then correlated to the presence of caffeic and rosmarinic acids [49]. Ferreres, et al., 2015 [50] reported the protective activity of *Mentha pulegium* infusions (which contained rosmarinic and salvianolic acids, as well as luteolin derivatives) in Caco-2 cells, where the extract protected against TBHP-induced cytotoxicity, increased total GSH and prevented GSSG accumulation [50].

Specifically, using *Thymus* spp. extracts, an ethanolic extract of *Thymus lanceolatus* (50 and 100 μg/mL) reduced TBHP-induced oxidative stress in Caco-2, SH-SY5Y (human neuroblastoma), and K562 (human chronic myelogenous leukaemia) cells [51]. The protective effect of an aqueous extract from *Thymus quinquecostatus* in Chang liver cells (which are now known to be derived from HeLa cells) by reducing TBHP-induced oxidative stress and cytotoxicity at 50, 100 and 200 μg/mL was reported [52].

**Figure 4:**
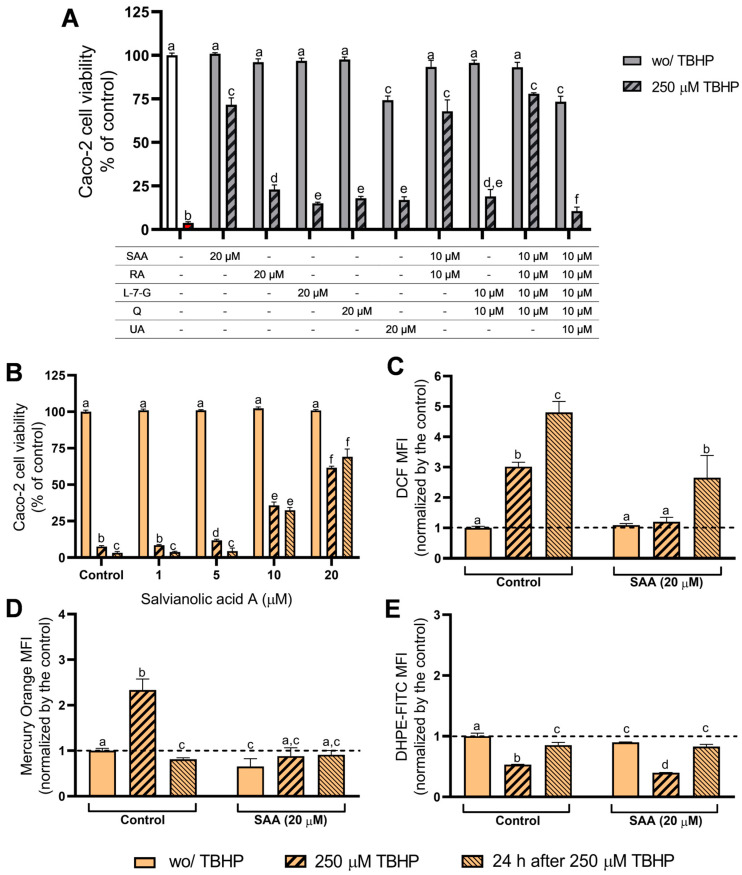
Effect of individual phytochemicals on cell viability and their action on antioxidant protection against TBHP insult. (**A**) Viability of Caco-2 cells exposed to individual phytochemicals and combinations of them and their capacity to inhibit TBHP-induced cytotoxicity. (**B**) Evaluation of SAA-induced cellular antioxidant protection against TBHP-induced oxidative insult. (**C**–**E**) Assessment of intracellular ROS (**C**), GSH content (**D**) and lipid peroxidation (**E**), using flow cytometry. A pre-exposure of Caco-2 cells to the phytochemicals for 4 h was used in all assays (see methods for details). Concerning statistical analysis, significant statistical differences (*p* < 0.05) between samples were denoted with different lowercase letters. All data was normalized to negative control (dotted line). Results are presented as mean ± SD (*n* = 3 independent experiments).

To explore the link between the extracts’ phytochemical composition and their cellular antioxidant activity, the present study also evaluated the cellular antioxidant activity produced by the main phytochemical compounds previously identified and quantified in the extracts [27,28], namely RA, SAA, L-7-G, quercetin and UA. The safety profile of these phytochemicals was previously addressed by Martins-Gomes, et al., 2024 [29], who reported that only UA induced cytotoxicity at 50 μM in Caco-2 cells (24 h exposure). Only AD extracts (which do not have triterpenoids) were further used to assess oxidative stress markers. Thus, the effect of UA was only analysed to highlight possible differences between the antioxidant activity of AD and HE extracts. As observed for *T. carnosus* extracts (Figure 1A), despite not reducing cell viability at 50 μM (24 h exposure), RA and quercetin induced cell death in Caco-2 cells as evaluated by Annexin/PI assay [29], thus a lower concentration was selected for this assay. Therefore, Caco-2 cells were pre-exposed (4 h) to individual phytochemicals (at 20 μM) and the results are presented in Figure 4A.

Of the tested compounds, UA was the only one that reduced cell viability (reduction of ~25%, at 20 μM), and provided minimal protection against TBHP-induced cytotoxicity (Figure 4A). As UA is among the main compounds in both *T. carnosus* and *T. capitellatus* HE extracts, and since the triterpenoid is not a main contributor to the antioxidant activity of HE extracts, and helps to explain the lower antioxidant potential of HE extracts when compared to AD extracts, despite the higher concentration in phytochemicals.

Mimicking the effect of AD extracts, a mixture containing 10 μM of SAA, RA, L-7-G and quercetin effectively reduced TBHP-induced cytotoxicity (cell viability of ~78%), in contrast to cells only exposed to TBHP (~4%), and the mixture per se did not reduce the cell viability (Figure 4A). However, when adding 10 μM UA to the mixture, simulating HE extracts, this mixture reduced cell viability to ~73%, identical to the cell viability of cells exposed to 20 μM of UA (~75%). In addition, this mixture conferred the lowest protection against the oxidative insult (cell viability ~11%; Figure 4A). Therefore, the cellular antioxidant effect promoted by these *Thymus* spp. extracts is dependent on the presence of phenolic compounds, while other components (such as triterpenoids) produce an opposing effect. Thus, the composition of extracts, which depends on extraction methods (AD vs. HE), justifies the differences observed between the AD and HE extracts.

Regarding the effect of phenolic compounds, phenolic acids induced higher cellular antioxidant activity than the flavonoids (Figure 4A). No significant differences were found between L-7-G and quercetin, regarding the ability to prevent oxidative damage. On the other hand, SAA induced a significantly higher cellular antioxidant effect than RA (*p* < 0.05). While in Caco-2 cells pre-exposed to RA, L-7-G or quercetin was observed cell viability >25% after TBHP exposure, in cells pre-exposed to SAA a cell viability of ~72% was observed, thus being this the compound that induces the highest correlation with the protective effect observed for the extracts. Aiming to understand whether the phytochemicals of the same class produced a cumulative effect, the simultaneous pre-exposure to 10 μM of SAA and RA (20 μM of phenolic acids), or to 10 μM of L-7-G and quercetin (20 μM of flavonoids) was analysed. The mixture of flavonoids produced the same effect as 20 μM of each individual flavonoid (Figure 4A). Regarding the phenolic acids, the mixture induced lower (but not significant, *p* > 0.05) protective activity than 20 μM SAA alone (Figure 4A). In addition, the mixture containing SAA, RA, L-7-G and quercetin (40 μM of polyphenols) was slightly more effective than 20 μM SAA alone (*p* < 0.05). Therefore, since SAA was the most effective phytochemical, a dose–response assessment of SAA in protecting Caco-2 cells against TBHP-induced oxidative damage (Figure 4B), and the effect of 20 µM SAA on the modulation of oxidative stress markers (Figure 4; panels C to E) were performed.

Pre-exposure of Caco-2 cells to SAA (1 to 20 µM) for 4 h did not induce cytotoxicity (Figure 4B). Other set of cells pre-exposed to SAA, were then treated with TBHP (250 µM; 4 h). The analysis of cell viability was assessed immediately after the exposure to TBHP, and a significant protective effect was observed at SAA concentrations ≥ 5 µM. Considering the additional recovery period of 24 h, a significant protective effect was observed at concentrations ≥ 10 µM. A dose–response effect was observed for both experimental conditions. As no cytotoxicity was induced by SAA, and since the highest concentration tested (20 µM) induced the highest protective effect, this concentration was chosen to evaluate intracellular oxidative stress markers.

As seen in Figure 4, pre-exposure of cells to 20 µM SAA did not change intracellular ROS content (Figure 4C), but reduced GSH content (Figure 4D), and slightly induced lipid peroxidation (Figure 4E). However, SAA effectively reduced the TBHP-induced rise in ROS (Figure 4C) and normalized GSH content (Figure 4D), but was unable to prevent lipid peroxidation in cells analysed after TBHP exposure (Figure 4E). However, when cells were allowed to recover for 24 h after the oxidative stimulus, intracellular ROS levels increased, albeit to a lesser extent than in the positive control cells (Figure 4C). Thus, SAA is effective against acute oxidative damage. However, considering cell survival at 24 h after TBHP exposure, it is clear that other phytochemicals in the extracts may contribute, as SAA was not able to maintain control-like ROS levels (Figure 4C).

**Figure 5:**
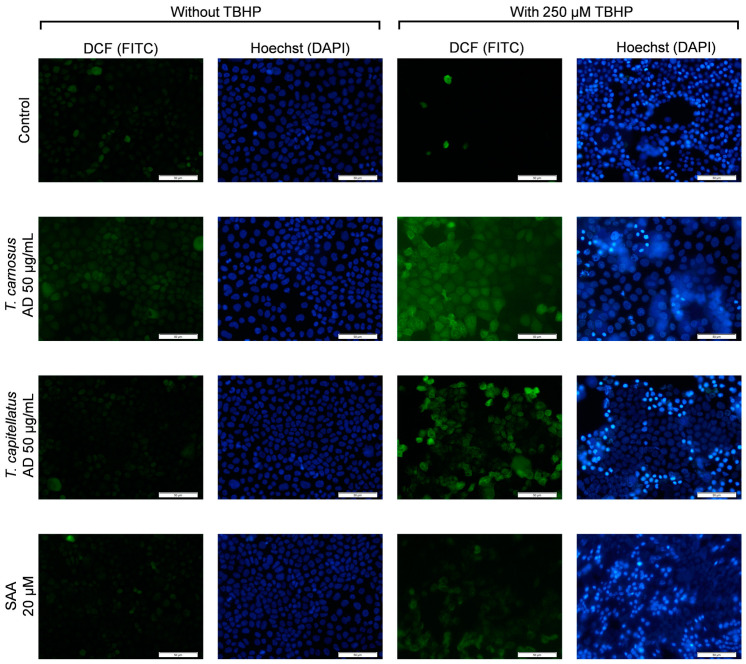
Fluorescence microscopy analysis of intracellular ROS (DCF) and DNA fragmentation (Hoechst 33342) in Caco-2 cells exposed to 250 µM TBHP and/or pre-exposed to extracts and SAA, as denoted. Images obtained using fluorescence microscopy and magnification of 100× (scale bar: 50 μm). See methods for details.

In order to confirm some of the results described previously, fluorescence microscopy was used to evaluate the ROS content in Caco-2 cells pre-exposed to extracts and SAA, before and after exposure to TBHP. DNA integrity was also evaluated, with the results presented in Figure 5.

Confirming the results obtained by flow cytometry, *T. carnosus* and *T. capitellatus* AD extracts, as well as SAA did not induce major changes in DCF fluorescence, and cells present control-like chromatin staining (Hoechst 33442), with a low number of cells presenting aberrant nucleus shape or high-fluorescence intensity (Figure 5).

In cells exposed only to 250 µM TBHP, the number of cells stained with DCF is scarce, despite many cells being stained with Hoechst 33342, revealing a cell count similar to the control (and also similar to brightfield images presented in Figure 2). Hoechst 33342 is a cell-permeable probe that binds to DNA independently of the cell’s metabolism, being used in both live and fixed cells [53]. On the other hand, DCFDA is a cell-permeable probe that once in intracellular environment is deacetylated by cytosolic esterases, forming a non-fluorescent intermediary compound that is then oxidized by intracellular ROS, producing the fluorescent moiety DCF [54]. Thus, DCF staining is dependent on cell metabolism, and we observed through Alamar Blue assay that less than 5% of the cells reduced resazurin (Figure 1). Thus, most cells that present positive Hoechst 33342 staining are not metabolically viable. And, the ones that present viability and DCF staining, in which a higher fluorescence is observed, implying higher ROS content, may reflect those 5% of cell viability.

Compared to Caco-2 cells exposed only to TBHP, those pre-exposed to *Thymus* spp. AD extracts before TBHP exposure presented a higher number of cells stained with DCF, indicating that a higher number of metabolically active cells exists, supporting the cell viability (Alamar Blue assay) results. In cells pre-exposed to SAA, a reduction in DCF staining was observed, with fluorescence intensity similar to that of non-exposed cells, also in line with the flow cytometry results (Figure 4C). Regarding DNA integrity, exposure of cells to TBHP induced an increase in Hoechst 33342 staining, revealing a higher percentage of cells with fragmented chromatin (Figure 5). Both the extracts and SAA reduced TBHP-induced DNA damage in Caco-2 cells (Figure 5).

**Figure 6:**
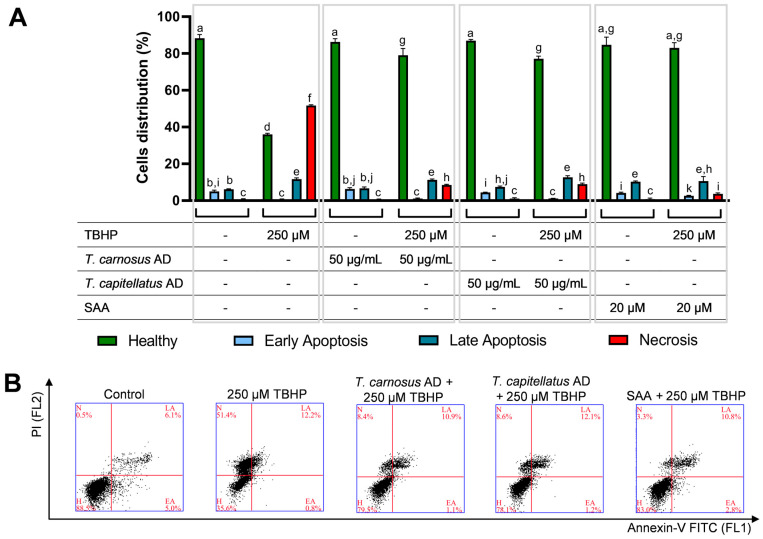
Prevention of TBHP-induced cell death by *Thymus* spp. AD extracts and SAA. (**A**) Evaluation of cell death through Annexin-V FITC/PI double staining assay using flow cytometry. (**B**) Examples of flow cytometry plots used for the cell death analysis presented in panel A (H: healthy; EA: early apoptosis; LA: late apoptosis; N: necrosis). Concerning statistical analysis, significant differences (*p* < 0.05) were denoted with different letters lowercase letters above each bar. Results are presented as mean ± SD (*n* = 3, independent experiments).

Depending on the concentration used, TBHP was shown to induce either apoptosis or necrosis. Lower concentrations induce apoptosis, while higher concentrations favour necroptosis, in both cases the process is mediated by an increase in oxidative stress [55]. Since we observed many cells with compromised DNA integrity (Figure 5), and loss of cell viability (Figure 1), we analysed the effect of TBHP (250 µM) in Caco-2 cells’ death mechanisms, and the protective effect induced by the extracts and SAA. Results are presented in Figure 6. It was observed that TBHP induced an increase in cells with Annexin^+^/PI^+^ staining, indicating late apoptosis or necroptosis, as well as with Annexin^−^/PI^+^ staining (Figure 6A), indicating necrotic cell death [56]. In cells only exposed to 250 µM TBHP, healthy cells (Annexin^−^/PI^−^ staining) percentage was reduced to ~36% (Figure 6). In cells pre-exposed to *T. carnosus* AD extract (50 µg/mL), *T. capitellatus* AD extract (50 µg/mL) or SAA (20 µM), but not to the oxidative agent, there was no significant reduction in healthy cell population percentage, confirming that the concentrations chosen are not cytotoxic.

The protective effect of both extracts and SAA against TBHP-induced cell death was notorious, as the percentage of healthy cells increased to ~79%, ~77% and ~83%, for *T. carnosus* AD extract (50 µg/mL), *T. capitellatus* AD extract (50 µg/mL) and SAA (20 µM), respectively, which was accompanied by a significant decrease of Annexin^−^/PI^+^ cells from ~51% (cells only exposed to TBHP) to ≤9% (Figure 6). Therefore, the *Thymus* spp. AD extracts can prevent TBHP-induced cell death, and in which SAA plays a predominant role.

In human retinal pigment epithelial (ARPE-19) cells, 390 µM TBHP induced mainly necrotic cell death, which was effectively reversed in cells pre-exposed to a standardized *Ginkgo biloba* extract (50 µg/mL) [40]. In Chang liver cells, *T. quinquecostatus* aqueous extract reduced the percentage of apoptotic cells induced by exposure to TBHP [52].

Also, in Caco-2 cells, rosmarinic acid (10 µM) was shown to reduce benzo[*a*]pyrene-induced mutagenicity and oxidative stress [57], luteolin reduced decabromodiphenyl ether (BDE-209)-induced cytotoxicity and oxidative stress [58], and pre-exposure to quercetin (10 µM) reduced FCCP- and oligomycin-induced increase in intracellular ROS [59].

Regarding the phytochemical with higher relevance in this work, Laka, et al., 2021 [60] showed that SAA (5 and 50 µM) was able to prevent H_2_O_2_-induced cytotoxicity in ARPE-19 cells, decreasing ROS levels and apoptotic events. The effect was shown to be mediated by Nrf2 and HO-1 activation, in a process dependent on the activation of Akt pathway [60]. In fact, the ability of natural products to mitigate oxidative damage in intestinal tract experimental models has been widely associated with the activation of Nrf2 pathway, and its downstream targets HO-1 and NQO1 [8].

### 3.3. Nrf2 Pathway as a Molecular Target of Extracts- and SAA-Induced Preventive Cellular Antioxidant Activity

**Figure 7:**
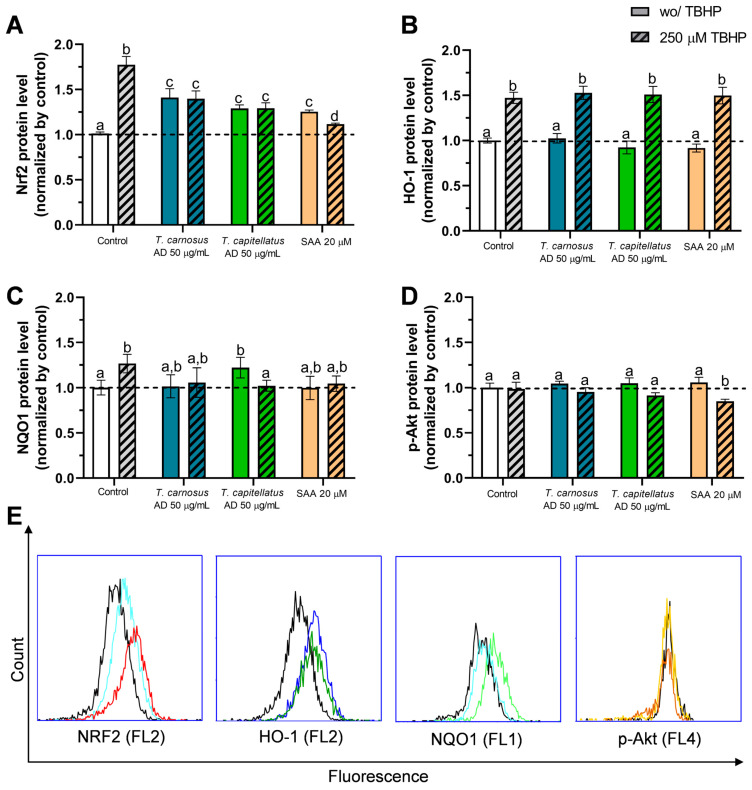
Effect of *T. carnosus* AD extract, *T. capitellatus* AD extract and SAA (as denoted) on antioxidant response related signalling pathways, in Caco-2 cells, evaluated using flow cytometry. (**A**) Nrf2 protein levels; (**B**) HO-1 protein levels; (**C**) NQO1 protein levels, (**D**) p-Akt protein levels**.** Significant statistical differences (when *p* < 0.05) were denoted with different lowercase letters above each bar. Results are presented as mean ± SD (*n* = 3 individual experiments). (**E**) Examples of flow cytometry plots used for the assessment of Nrf2, HO-1, NQO1 and p-Akt protein levels; Lines denoted in black: control (non-exposed cells); red: 250 µM TBHP (4 h); light blue: pre-exposure to *T. carnosus* AD (50 µg/mL; 4 h); light green: pre-exposure to *T. capitellatus* AD (50 µg/mL; 4 h); dark blue: pre-exposure to *T. carnosus* AD (50 µg/mL; 4 h) and then to TBHP (250 µM; 4 h); dark green: pre-exposure to *T. capitellatus* AD (50 µg/mL; 4 h) and then to TBHP (250 µM; 4 h); light orange: pre-exposure to SAA (20 µM; 4 h); dark orange: pre-exposure to SAA (20 µM; 4 h) and then to TBHP (250 µM; 4 h). Data from fluorescence channels 1, 2 and 4 (FL1, FL2 and FL4) are presented in logarithmic scale. All data was normalized to negative control (dotted line).

Aiming to understand the molecular mechanisms behind the cellular antioxidant activity observed above, the role of Nrf2, HO-1 and NQO1 in the effects observed in Caco-2 cells induced by extracts of *Thyme* spp. extracts and SAA were evaluated. In Figure 7, it is observed that the exposure of Caco-2 cells to 250 µM TBHP resulted in increased protein levels of Nrf2 (1.77-fold), HO-1 (1.47-fold) and NQO1 (1.27-fold). The Nrf2 pathway is a well-described intervenient in oxidative stress response [61].

In homeostasis, Nrf2 is sequestered by Keap1 (Kelch-like ECH-associated protein 1) in the cytoplasm, which promotes its ubiquitination and further degradation [62]. Under oxidative stress, oxidation of specific cysteine residues on Keap1 promotes Nrf2 release and its translocation to the nucleus, where it triggers the expression of genes encoding endogenous antioxidant enzymes, HO-1, NQO1, as well as enzymes involved in GSH synthesis [61,63,64].

In Caco-2 cells pre-exposed to *Thymus* spp. AD extracts, Nrf2 protein levels were increased, and the effect was, at least partially, mediated by SAA, as it induced a similar effect (Figure 7A). Comparing extracts, *T. carnosus* AD extract induced the highest increase in Nfr2 protein level (1.41-fold), however differences are not statistically significant from *T. capitellatus* AD extracts. Cells pre-exposed to the extracts, and then treated with TBHP, showed a significant reduction in Nrf2 protein level, comparing to TBHP control (*p* < 0.05), being Nrf2 levels similar to that of cells exposed only to extracts (Figure 7A). Cells pre-exposed to SAA also prevented TBHP-induced increase in Nrf2 protein level (1.12-fold; Figure 7A). Thus, it is demonstrated that Nrf2 plays a significant role in the cellular antioxidant action of *T. carnosus* and *T. capitellatus* AD extracts, with a significant contribution of SAA.

Regarding the downstream targets of Nrf2, neither AD extracts nor SAA induce significant changes in HO-1 levels (*p* > 0.05), thus implying that HO-1 does not have significant role in the cellular antioxidant effect observed. However, HO-1 levels increase in cells treated with TBHP (Figure 7B). Regarding NQO1, exposure of cells to *T. carnosus* AD extract or to SAA, did not change basal NQO1 levels, while *T. capitellatus* AD extract induced a significant increase in NQO1 protein level (1.22-fold). However, both extracts and SAA effectively reduced TBHP-dependent NQO1 protein expression (Figure 7C). Thus, while both AD extracts increased Nrf2 protein level, only *T. capitellatus* AD extract was able to increase NQO1 protein level, thus implying different mechanisms behind the cellular antioxidant activity.

In Figure 3, it was observed that Caco-2 cells pre-exposed to *T. capitellatus* AD extract presented a slight increase in intracellular ROS and GSH content, unlike that for exposure to *T. carnosus* extract. Also, while SAA prevented oxidative damage through Nrf2 increase, but not NQO1, its effect is more closely correlated to *T. carnosus* AD extract, while other phytochemicals in *T. capitellatus* AD extract should induce NQO1 expression (Figure 7). Under oxidative stress, the flavoprotein NQO1 mediates the reduction of quinones to hydroquinones, acting directly in superoxide radical scavenging, providing physiological antioxidant forms of vitamin E and ubiquinone, it is also an intracellular generator of NAD^+^, and participates in protein stabilization and translation [65,66]. Using rat small intestine epithelial cells (IEC-6), a dried root extract of *Astragalus membranaceus* was shown to activate Nrf2 translocation to the nucleus and induce NQO1 protein expression [67]. In a different study, the isoflavone puerarin was shown to upregulate Nrf2 and NQO1 protein expression, preventing dextran sulfate sodium-induced oxidative stress in the colon of BALB/c mice [68]. In other experimental models, salvianolic acid A induced the activation of Nrf2/HO-1 evaluated in the intestinal tissue of Sprague-Dawley rats with intestinal ischemia-reperfusion injury [69], as well as in ARPE-19 cells subjected to H_2_O_2_-induced oxidative stress [70]. Rosmarinic acid activated Nrf2/NQO1 pathway to counter H_2_O_2_-induced oxidative stress, in human liver cells (L02 cells) [71]. In HepG2 cells, quercetin induced the expression of *NQO1* and other antioxidant response elements through Nrf2 activation [72].

Regarding Nrf2 activation, it was reported that PI3K (phosphoinositide 3-kinases)/Akt pathway is an effective activator of Nrf2 signalling cascade [73]. In mouse Sertoli cells, resveratrol mitigated zearalenone-induced oxidative damage via Nrf2 and HO-1 activation, which was mediated by PI3K/Akt pathway [74]. Thus, in order to analyse if the induction of Nrf2 activation by the selected *Thymus* extracts and SAA was through Akt modulation, we analysed the content in p-Akt (the activated form of Akt, mediated by PI3K signalling [75]) and results are presented in Figure 7D.

Regardless of the exposure of Caco-2 cells to TBHP, or pre-exposure of cells to extracts or SAA, we did not observe a significant increase in p-Akt protein levels (Figure 7D, *p* > 0.05). However, a significant decrease in p-Akt content (*p* < 0.05) is observed in cells pre-exposed to SAA with further challenge with TBHP, while a non-significant decrease (*p* > 0.05) is observed in cells pre-exposed to the extracts with further challenge with TBHP. It should also be considered that p-Akt is not the only upstream inducer of Nrf2.

In addition, various phytochemicals have been shown to induce pro-survival effects in cells exposed to oxidative agents in addition to the antioxidant response, by inhibiting pro-apoptotic proteins and promoting the expression of pro-survival proteins [76]. This is the case of RA, which reduced H_2_O_2_-induced apoptosis in SH-SY5Y cells, by downregulating caspase-3 and Bax, and upregulating Bcl-2 and PI3K [77]. SAA also reduced H_2_O_2_-induced apoptosis in ARPE-19 cells, but via mTOR (mammalian target of rapamycin) upregulation, a molecular target also dependent on PI3K/Akt pathway [70]. Therefore, the analysis of mTOR, as well of pro- and anti-apoptotic effectors may provide new insights in the protective effect of *T. carnosus* and *T. capitellatus* AD extracts against oxidative damage and will be the focus of future research. Regarding the thyme species studied in the present work, to the best of our knowledge this is the first report on their cellular antioxidant activity and respective molecular targets. Although it was observed that the protective antioxidant effect induced by AD extracts and SAA is related to increased Nrf2 protein levels, a further analysis of specific interactions with this pathway and additional molecular targets are needed. Concerning Nrf2 pathway, future studies should address whether AD extracts and SAA increase *NFE2L2* expression (the gene that encodes Nrf2), and/or promote Nrf2 translocation to the nucleus, and evaluate protein and mRNA expression of other upstream activators and downstream targets of Nrf2.

Regarding other studies reporting thyme extracts in different experimental models, the protective effect of an ethanolic extract of *T. quinquecostatus* in cerebral ischemia–reperfusion (evaluated in Sprague Dawley rats) was shown to be dependent on Nrf2 and HO-1 activation [78]. An ethanolic extract of *T. vulgaris* protected normal human dermal fibroblasts against ultraviolet radiation, in an effect mediated by Nrf2 activation with further increased protein expression of HO-1 and NQO1 [79]. Thus, natural plant extracts and their individual compounds demonstrate an important role in protecting cells against oxidative, and other environmental, damaging agents, and warrant further investigations.

## 4. Conclusions

In this study, we explored the potential of two *Thymus* spp. extracts as dietary antioxidant agents. While most studies report the use of ethanolic/methanolic extracts, our findings demonstrate that aqueous extracts offer a higher potential for regulating the intestinal epithelium redox state, in addition to lower cytotoxicity. This result is particularly relevant, as aqueous extracts mimic the typical consumption method of thyme. In addition, a major constituent of *T. carnosus* and *T. capitellatus* extracts, SAA, also induced significant cellular antioxidant activity. Given that HE extracts contain higher concentrations of salvianolic acids, future studies should consider this extraction method when using these species as sources of nutraceuticals. Antioxidant activity was observed with AD extracts and SAA at concentrations of 50 µg/mL and 20 µM, respectively. Cells pre-exposed to AD extracts and SAA presented reduced intracellular ROS levels, reduced DNA damaged, and decreased percentage of cells with morphological changes. In Caco-2 cells pre-exposed to the extracts, control-like or improved levels of oxidative stress markers were observed 24 h after the oxidative stimulus. In addition, both extracts and SAA reduced TBHP-induced cell death, with this effect being partly mediated by Nrf2 activation in both extracts and NQO1 in cells pre-exposed to *T. capitellatus* AD extract. This study is the first to assess the potential of *T. capitellatus* and *T. carnosus* as dietary antioxidant agents, shedding light on their cellular targets.

## Data Availability

The original contributions presented in the study are included in the article, further inquiries can be directed to the corresponding author.
